# The PI3K/AKT/mTOR and CDK4/6 Pathways in Endocrine Resistant HR+/HER2− Metastatic Breast Cancer: Biological Mechanisms and New Treatments

**DOI:** 10.3390/cancers11091242

**Published:** 2019-08-24

**Authors:** Daniele Presti, Erica Quaquarini

**Affiliations:** 1 Department of Internal Medicine and Therapeutics, University of Pavia, 27100 Pavia, Italy; 2 Medical Oncology Unit, IRCCS ICS Maugeri SpA SB, 27100 Pavia, Italy; 3 Experimental Medicine, University of Pavia, 27100 Pavia, Italy

**Keywords:** metastatic breast cancer, endocrine resistance, PI3K, CDK4/6 inhibitors, mTOR

## Abstract

Endocrine-based treatments are the normal standard-of-care in women with hormone receptor-positive/Human Epidermal growth factor Receptor 2-negative metastatic breast cancer. Despite the well-known efficacy of these drugs as first-line therapies, about 50% of women develop endocrine resistance and disease progression. The treatment of these patients has represented one of the most important research fields in the last few years, with several multicenter phase II/III trials published or still ongoing. Novel therapies, such as cyclin-dependent kinase (CDK)4/6 and phosphatidylinositol 3-kinase (PI3K)/protein kinase B (AKT)/mammalian target of rapamycin (mTOR) inhibitors, have significantly changed the prognosis of patients progressing to a previous endocrine treatment, allowing a great benefit in terms of progression-free survival and, in some cases, of overall survival. However, identifying response predictors is essential for the rational use of these drugs to avoid unnecessary toxicity and costs, and to ensure the optimal therapeutic sequence is used. In this review, we analyze the PI3K/AKT/mTOR and CDK4/6 pathways and their roles in endocrine resistant metastatic breast cancer. We then focus on the new treatments developed and the roles of these drugs in overcoming endocrine resistance, describing the latest clinical trials that led to the approval of the drugs in clinical practice.

## 1. Introduction

Breast cancer (BC) is the most frequent neoplasm among women, and it is classified into four different subtypes based on the estrogen receptor (ER), progesterone receptor (PgR), and Human Epidermal growth factor Receptor 2 (HER2) expression [1]. About 75% of BCs express ER and/or PgR, and this fact is indicative of hormone dependency. Despite progress in treatment strategies, metastatic breast cancer (MBC) remains an incurable disease, with a median overall survival (OS) of 3 years and a 5-year survival rate of 25% [2]. Endocrine-based treatments, such as selective ER modulators (tamoxifen), selective ER down-regulators (fulvestrant), and aromatase inhibitors (AIs), are established standards of care in women with hormone receptor (HR)+/HER2− MBC. The choice between these regimens depends on the type and duration of adjuvant endocrine therapy used as well as the time elapsed from the end of adjuvant endocrine therapy. In all pre-menopausal women, ovarian follicle suppression or ovarian follicle analogue agents should be associated [2]. Besides the well-known efficacy of these treatments as first-line therapies in women without visceral crisis, about 50% of patients develop endocrine resistance leading to therapeutic failure [3]. Primary endocrine resistance is defined as relapse during the first 2 years of adjuvant endocrine therapy or progressive disease within the first 6 months of first-line endocrine therapy for MBC [2]. Secondary resistance is present (1) when a relapse occurs after the first 2 years of adjuvant endocrine therapy; (2) when a relapse occurs within 12 months of completing adjuvant endocrine therapy; or (3) when a progressive disease occurs after more than 6 months from the beginning of endocrine therapy for MBC. In this context, both combination and sequential single chemotherapy agents are alternative options [2]. In particular, monotherapy should be the preferred choice and combination treatments should be given to patients with rapid disease progression, visceral crisis, or the need for symptom control. The chemotherapy regimen should be chosen according to the (neo)adjuvant therapy performed and should take into account the different toxicity profiles and patient preferences. Anthracyclines and taxanes are usually preferred in patients who have not received these regimens as (neo)adjuvant treatments. Other options are capecitabine, vinorelbine, eribulin, gemcitabine, platinum agents, and liposomal anthracyclines. In recent years, the addition of a cyclin-dependent kinase (CDK)4/6 inhibitor or everolimus to endocrine therapy has represented a significant practice-changing goal that has led to the use of chemotherapy being postponed [2]. Recently, the Young-PEARL trial presented by Park and colleagues at the American Society of Clinical Oncology (ASCO) 2019 Annual Meeting further stressed the superiority of an endocrine regimen plus a CDK4/6 inhibitor over chemotherapy in patients affected by HR+/HER2− MBC and pre-treated with one chemotherapy line [4]. In this review, we analyze the phosphatidylinositol 3-kinase (PI3K)/protein kinase B (AKT)/mammalian target of rapamycin (mTOR) and CDK4/6 pathways and their roles in endocrine-resistant HR+/HER2− MBC. We then focus on the newly developed treatments and the roles of these drugs in overcoming endocrine resistance, describing the latest clinical trials that led to the approval of the drugs in clinical practice.

## 2. Biological Mechanisms behind Endocrine Resistance

**Figure 1:**
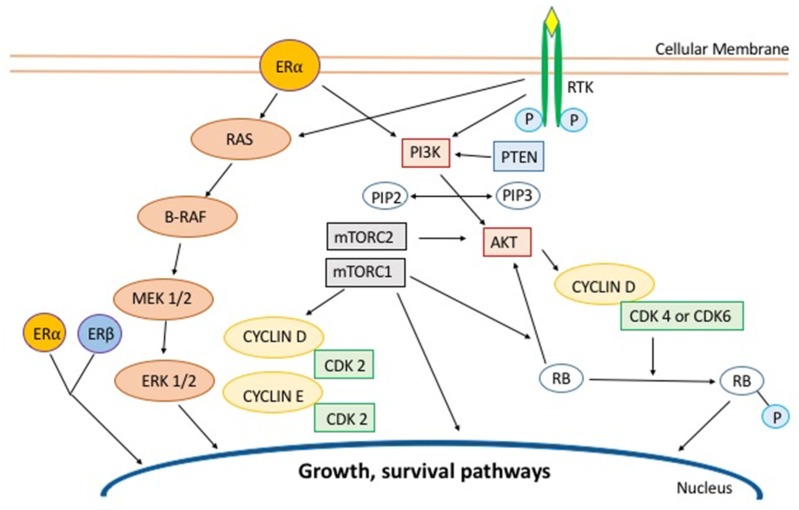
Intracellular growth factors and signals involved in breast cancer cell replication: Several proteins, transcription factors, and soluble mediators have roles in the cascade of events that finally leads to cell replication, as described in each section of this review. The same mechanisms are involved in endocrine resistance. The estrogen receptor (ER)α and ERβ receptors contain two transactivation domains (activation function 1, AF1, and activation function 2, AF2). Upon binding of estradiol (E2) to ER, a series of successive triggers results in the translocation of chaperone proteins from the ERα, receptor dimerization, phosphorylation, and the subsequent binding of ER to the DNA. While AF2 serves as a binder for coactivators and corepressors, AF1 is not dependent on a ligand and its activation is mediated by crosslinks and crosstalk among the RAS/B-RAF, phosphatidylinositol 3-kinase (PI3K)/protein kinase B (AKT), and cyclin-dependent kinase (CDK)2 pathways. Moreover, activation of the growth factor receptor tyrosine kinases leads to the phosphorylation of the ER through the RAS/B-RAF and PI3K/AKT pathways. The CDKs are cyclin D -dependent drivers in the cell cycle and division. During the DNA synthesis phase of the cell cycle, the cyclin D-CDK4/6 complex serves as the enzyme that catalyzes the phosphorylation of the retinoblastoma (RB) protein and dictates DNA replication [5,6,11,14]. mTOR: mammalian target of rapamycin.

Several mechanisms are responsible for endocrine resistance, including the deregulation of multiple components of the ER/PgR pathway (aberration in ER expression, over-expression of ER co-activators, and down-regulation of co-repressors), altered regulation of signaling molecules involved in cell cycle or cell survival, and the activation of escape pathways that can provide cell replication (Figure 1) [5]. The ER is a nuclear receptor that comprises two subdivisions: ERα and ERβ. In many breast cancers, ERα activation by estrogens is considered to be responsible for enhanced proliferation. ERβ has contrasting effects to ERα and inhibits the stimulatory effects of estradiol (E2) on cell proliferation. Both receptors contain two transcription domains, activation function 1 (AF1) and activation function 2 (AF2), with AF1 being responsible for constitutive ER activity [6]. When E2 binds to ER, it activates a series of processes which lead to its binding as a dimer to specific sites on DNA, known as E2-response elements (EREs), leading to the transcription of target genes regulated by the synergistic activity of AF2 and AF1 [7]. The AF1 domain is not dependent on a ligand, and it regulates gene transcription, even in cells with an ERα deletion [8]. In this independent mechanism, the activation of AF1 is mediated by crosslinks and crosstalk among the RAS/RAF/MEK/ERK pathway (also known as mitogen activated protein kinase, MAPK), PI3K/AKT, and cyclin-dependent kinase 2/7 (CDK2/7) pathways. This mechanism might lead to resistance against various endocrine therapies [9]. Molecularly, estrogen activity can induce the activation of insulin-like growth factor and the PI3K/AKT and MAPK pathways, which can downregulate the expression of ER and PgR on the cell surface [10]. The PI3K/AKT/mTOR pathway is the most frequently altered in MBC, and the upregulation of these molecules promotes dependent and independent ER transcriptional activity, which contributes to anti-estrogen resistance, leading to tumor cell growth, survival, motility, and metabolism [11]. Drugs targeting this pathway showed promising results in combination with AIs or anti-estrogens in HR+/HER2− MBC. Furthermore, it has been demonstrated in vivo that PI3K and mTOR inhibition can even restore sensitivity to endocrine therapy, providing a strong rationale for the combination of the two therapies [12]. The PI3K/AKT/mTOR pathway, like other mitogenic pathways, such as the MAPK, the nuclear factor kappa-light-chain-enhancer of activated B cells/IκB kinase (NF-kB/IKK) and the ERs, can also provide the interaction between cyclin D and CDK4/6 [11]. The CDKs are cyclin D-dependent drivers of cell cycle and division. During the DNA synthesis phase of the cell cycle, the cyclin D-CDK4/6 complex leads to the hyperphosphorylation of the retinoblastoma (RB) protein, inactivating its growth inhibitory function by decoupling it from E2F transcription factors [13]. The pathway causes progression from the G1 to the S phase of the cell cycle. CDK4/6 is also regulated by the Inhibitor of Cyclin-Dependent Kinase 4 (INK4) family [14]. INK4 proteins, which include p16 INK4A, p15 INK4B, p18 INK4C, and p19 INK4D, can bind to both monomeric CDK, preventing its association with a cyclin, and the CDK-cyclin complex, forming an inactive ternary complex. In this way, the INK4 proteins weaken the binding of D-type cyclins to CDK4/6 and interact with the catalytic domains of CDK4/6 to suppress their kinase activity. These proteins can also negatively regulate CDK4 and CDK6 in response to stress conditions associated with cellular ageing or induced by multiple oncoproteins. The overexpression of p16 INK4A ultimately engages RB to suppress growth and cell cycle progression and promotes oncogene-induced senescence [14]. The dysregulation of INK4 has been associated with poor response to endocrine therapy [15]. Moreover, it has been observed that the loss of the tumor suppressor gene *Cyclin Dependent Kinase Inhibitor 2A* (*CDKN2A*), which encodes for p16 INK4A, is one of the most common abnormalities in BC, causing uncontrolled activation of CDK4/6, which leads to abnormal cell proliferation [15,16]. Everolimus, an mTOR inhibitor, was, in combination with AI, the first target drug developed with the aim of overcoming endocrine resistance [17]. Afterwards, the use of pan-PI3K inhibitors, in combination with endocrine therapy, showed low clinical benefit and response in HR+/HER2− MBC treatment and was associated with severe toxicity [18]. On the other hand, recent data showed that p110α-specific PI3K inhibitors have a better safety profile. In recent years, phase III clinical trials have also demonstrated the activity and efficacy of therapies targeting the CDK4/6 pathway [2,19]. In addition, recent data showed that CDK4 plays a key role in the hormone-independent cell growth mechanism [20].

## 3. PI3K/AKT/mTOR Pathway

### 3.1. mTOR

#### 3.1.1. From Biology to Drug Development

mTOR is a serine/threonine protein kinase, a downstream effector of AKT, that comprises two functionally different complexes: mTOR complex 1 (mTORC1) and mTOR complex 2 (mTORC2) [21]. mTORC1 belongs to a complex network of regulatory feedback loops, and, once activated, is responsible for limiting the proliferative signals transmitted by upstream effectors like platelet derived growth factor receptors α and β, which leads to the attenuation of PI3K/AKT activity. mTORC2, instead, regulates AKT phosphorylation at S473 and organizes the cellular actin cytoskeleton. In addition, mTORC1 activation leads to the direct reduction of mTORC2 activity and mTOR’s downstream substrate S6 kinase can phosphorylate and activate the functional domain of the ER, leading to ligand-independent receptor activation [21]. Everolimus, a rapamycin analogue that targets mTORC1, was the first drug to be approved for the treatment of patients affected by HR+/HER2− MBC and has progressed to a first-line therapy with an AI [22].

#### 3.1.2. Clinical Trials

In a phase II trial involving postmenopausal women with HR+/HER− MBC, 111 patients were randomized 1:1 to receive tamoxifen or tamoxifen plus everolimus [22]. The primary endpoint was the clinical benefit rate (CBR) that resulted higher in the combination arm (61% versus 42.1%, for the experimental and control arms, respectively). The most common adverse events (AEs) in the combination arm were fatigue (72%), stomatitis (56%), rash (44%), anorexia (43%), and diarrhea (39%) [23]. The study that led to everolimus approval was the BOLERO-2 trial, a phase III randomized, double-blinded study, in which 724 postmenopausal women with HR+ MBC were randomized to receive everolimus plus exemestane or exemestane plus placebo (Table 1) [22]. All patients developed progression during or following nonsteroidal aromatase inhibitor therapy. Randomization was stratified according to the presence of visceral metastases and sensitivity to prior endocrine therapy. The study met its primary endpoint of progression-free survival (PFS), which was higher in the experimental arm (11.0 versus 4.1 months, respectively; *p* < 0.0001). Secondary endpoints also favored the everolimus arm with an overall response rate (ORR) of 12.6% versus 1.7% (*p* < 0.0001) and a CBR of 51.3% versus 26.4% (*p* < 0.0001). The most commonly reported AEs with everolimus treatment included stomatitis (59%), rash (39%), fatigue (37%), and diarrhea (34%). Half of the patients experienced grade 3–4 AEs, among which stomatitis (8%), anemia (7%), hyperglycemia (5%), and fatigue (4%) were the most common. However, everolimus did not demonstrate an improvement in OS (31.0 versus 26.6 months in the experimental and placebo arms, respectively, *p* = 0.1426). Even though the trial was not powered to demonstrate an increase in OS, this data could suggest an early onset of drug resistance over the disease course. Moreover, 10–15% patients showed intrinsic resistance to mTORC1 inhibition [24]. A retrospective, exploratory analysis of the BOLERO-2 study evaluated genetic variations of cancer-related genes using next-generation sequencing (NGS) from archival tissues [25]. Individual evaluation of the four most commonly mutated genes (*PIK3CA*; *cyclin D1*; *checkpoint gene p53*; and *fibroblast growth factor receptor 1*, *FGFR1*) showed that PFS benefit in the everolimus arm in the presence of an alteration in one of these pathways was similar to the PFS benefit seen in the overall study population. However, patients with wild-type (WT) genes or only a single genetic alteration in the *PIK3CA*, *cyclin D1*, or *FGFR1* and *2* genes had greater benefit from everolimus [25]. Another retrospective trial determined the prevalence of estrogen receptor 1 (ESR1) mutations (Y537S and D538G) in cell-free DNA (cfDNA) baseline plasma samples from 541 out of 724 patients in the BOLERO-2 trial [26]. About 28.8% of samples had the ESR1 mutation D538G (21.1%) and/or Y537S (13.3%), while 30% had both. These mutations were found to be associated with shorter OS (WT, 32.1 months; D538G, 25.99 months; Y537S, 19.98 months; both mutations, 15.15 months). The D538G group derived a similar PFS benefit to that of WT patients from everolimus therapy. Outside of the rigorous inclusion/exclusion criteria of a randomized clinical trial, the EVA real-life study collected data on the efficacy and safety of the everolimus plus exemestane combination in a real-life setting [27]. In the study, 404 patients were enrolled. The primary endpoint was the evaluation of the median duration of everolimus treatment. Secondary endpoints included the ORR, disease control rate (DCR), and toxicity. The median everolimus dose intensity was >7.5 mg/day in most patients (59.4%). The median treatment duration was 31.0 weeks (range 15.4–58.3 weeks) in the population as a whole, and 23.3, 33.8, and 32.5 weeks, respectively, in the three dose intensity groups. No differences in regard to the dose intensity or type of previous treatment were described. The ORR and DCR were observed in 31.6% and 60.7% of the patients, respectively. Grade 3–4 AEs were reported in 37.9% of cases, among which stomatitis (11.2%), non-infectious pneumonitis (3.8%), anemia (3.8%), and fatigue (3.2%) were the most frequent ones. Recently, in the phase II MANTA study, vistusertib, an oral dual inhibitor of mTORC1 and mTORC2, was investigated [28]. In this study, postmenopausal women were eligible if they had disease recurrence while or within 12 months of the end of adjuvant treatment with AI or progression during or within one month of the end of AI treatment for locally advanced BC or MBC. A total of 333 patients were randomized 2:3:3:2 to receive fulvestrant alone, fulvestrant plus daily vistusertib (50 mg twice a day), fulvestrant plus intermittent vistusertib (2 days on, 5 days off; 125 mg twice a day), or fulvestrant plus everolimus (10 mg day). The primary endpoint was PFS, but no significant differences between the arms were seen, with the exception of improved PFS in the fulvestrant plus everolimus arm compared to fulvestrant plus vistusertib, and fulvestrant plus everolimus compared to fulvestrant alone.

#### 3.1.3. Mechanisms of Endocrine Resistance during mTOR Inhibition

In the aforementioned trials, about 50% of patients did not receive clinical benefit from everolimus treatment in the presence of hormone-resistant HR+/HER2− MBC. Emerging evidence suggests the heterogeneous activity of mTORC1 in advanced tumors as being an important limiting factor for the efficacy of everolimus, and several elements may contribute including genetic and functional heterogeneity as well as tumor hypoxia [29]. Many studies have proven that mTORC1 activity is largely reduced in tumor hypoxic regions, suggesting that tumor regions with low levels of oxygen use alternative pathways in order to grow and replicate [29]. Since hypoxic tumor cells actively participate in tumor progression, it is suggested that tumor regions displaying low levels of oxygen grow independently from mTORC1, and they are therefore insensitive to mTORC1 inhibition [30]. Consistent with this hypothesis, it was found that rapamycin reduced cancer cell proliferation in non-hypoxic tumor area had no effect on hypoxic tumor regions, prompting the proposal that mTORC1 inhibitors may exert a tumor-region-selective anti-proliferative effect [29]. Recently, in BC cells, survivin, a downstream target of the PI3K/AKT/mTOR pathway, was demonstrated to be involved in drug resistance, especially to taxanes and kinesin inhibitors [31]. Taglieri and colleagues investigated whether survivin was involved in the acquired resistance to everolimus treatment in hormone-sensitive breast tumors, showing that the drug is able to modify survivin expression in vitro in opposite ways with a downregulation in the sensitive cells and an upregulation in the resistant ones [32]. Moreover, the block of survivin upregulation in these cells restored sensitivity to everolimus, suggesting that survivin is a potential target for future treatments [32]. The acquired resistance to mTORC1 inhibitors was attributed to compensatory feedback loops involving the activation of oncogenes such as *Myc*, the upregulation of which is mediated by the transcriptional regulator bromodomain-containing protein 4 (BRD4) [33]. Recently, Kimura and colleagues studied everolimus-resistant cells generated from two cell lines resistant to AIs: the first had upregulated PI3K/AKT/mTOR signaling and constitutive ER overexpression; the second had low ER expression and upregulated receptor tyrosine kinase/c-jun N-terminal kinase (RTKs/JNK) signaling [34]. Both cell lines received long-term exposure to everolimus in vitro. The study showed that, even if acquired resistance to everolimus did not affect ER expression, it was able to de-regulate ER signaling by downregulating the PgR and ER1 pathways. However, cells that lost ER expression gained everolimus resistance faster, suggesting that ER expression and signaling might delay resistance to mTORC1 inhibitors. This study also tried to investigate whether chemotherapy agents could be effective against everolimus-resistant cells, but no positive results were found. 

### 3.2. PI3K/AKT

#### 3.2.1. The Intracellular Molecular Pathway

The PI3K protein family comprises three classes of lipid kinase that catalyze the phosphorylation of phosphatidylinositol 4,5-bisphosphate (PIP2) to phosphatidylinositol 3,4,5-trisphosphate (PIP3) [11]. The PI3K class 1 family, a heterodimer composed of a regulatory and a catalytic subunit, is the most frequently implicated in human cancer [35]. Somatic mutations in genes encoding for components of the PI3K pathway occur in more than 70% of BCs. They include mutations or amplification of PIK3CA (subunit p110α), PIK3CB (subunit p110β), and PIK3R1 (subunit p85α), which are the catalytic subunits of PI3K, or mutations of PI3K modulators, such as PTEN, AKT, and mTOR [21,35]. The most common mechanism of PI3K pathway activation is through mutations or amplifications of PIK3CA, where p110α plays an important role in the tumorigenesis of BC though extra-nuclear ER signaling, and is often responsible for endocrine treatment resistance [36]. Indeed, PIK3CA mutation occurs in almost 40% of HR+/HER2− MBC, and it usually appears early in breast tumor progression [37]. Therefore, inhibition of the PI3Kα isoform has been studied as a possible new strategy in order to overcome endocrine resistance. 

#### 3.2.2. Clinical Trials

Preclinical and clinical studies showed that PI3K/AKT/mTOR pathway inhibition has a synergistic effect with endocrine therapies, and could reduce tumor progression in HR+ PI3K mutant BCs beyond the first endocrine line of treatment [34,38,39]. The BELLE-2 trial was a phase III, double-blind, placebo-controlled randomized study that included women with HR+/HER2− MBC, whose diseases progressed on or after endocrine treatment with an AI (Table 1) [40]. In total, 1147 patients were randomly assigned to receive fulvestrant plus buparlisib, a panPI3K inhibitor, versus fulvestrant plus placebo. The trial met its primary endpoint since it demonstrated an improvement in PFS for the experimental arm (6.9 versus 5.0 months, respectively, *p* = 0.00021). However, an exploratory analysis in patients with PIK3CA mutation (detected in circulating tumor DNA, ctDNA) described a benefit in terms of PFS from panPI3K inhibition (7.0 versus 3.2 months for the experimental and control arms, respectively, *p* not available), although patients without the PI3KCA mutation did not receive any additional benefit from buparlisib (6.8 months for both arms). Regarding OS, a survival improvement in favor of the buparlisib arm was described, but it did not result in statistical significance (33.2 versus 30.4 months, for the experimental and control arm, respectively). Furthermore, among PIK3CA-mutant ctDNA patients, the OS benefit was not statistically significant (26.0 versus 24.8 months) [41]. The most common grade 3–4 AEs in the buparlisib group were increases in alanine aminotransferase and aspartate aminotransferase (25% versus 18%, respectively), hyperglycemia (15%), and skin rash (8%) [40,41]. Another panPI3K inhibitor, pictilisib, was tested in association with fulvestrant in a phase II, placebo-controlled, randomized clinical trial in which postmenopausal women with HR+/HER2−MBC, resistant to a preceding treatment with an AI in adjuvant or metastatic setting, were enrolled (the FERGI trial) (Table 1) [42]. The study was divided into two parts: part 1 included patients with or without PIK3CA mutations, whereas part 2 included only patients with PIK3CA mutations. In total, 168 and 61 patients were randomly allocated to parts 1 and 2, respectively. The primary endpoint was PFS; PIK3CA mutation was assessed by quantitative real-time PCR. The study did not reach its primary endpoint, since no differences in terms of PFS were seen between the pictilisib arm and the placebo one in part 1 (6.6 versus 5.1 months for the experimental and control arms, respectively, *p* = 0.096). Also, in part 2, no differences were evident (5.4 versus 10.0 months, respectively, *p* = 0.84). Regarding safety, in part 1, grade 3 or worse AEs occurred in 61% of patients in the pictilisib group, 16% of which were serious AEs. In part 2, grade 3 or worse AEs occurred in 36% of patients in the pictilisib arm with 5% having serious AEs. The most common toxicities were maculopapular rash, diarrhea, fatigue, and elevated alanine and aspartate aminotransferase levels. Interestingly, the AEs of buparlisib and pictilisib were related to their low selectivity for the PI3K pathway, suggesting that more selective PI3K inhibitors could improve tolerability [41,42,43]. Alpelisib was the first oral inhibitor that selectively targets the PI3Kα isoform to demonstrate antitumor activity in HR+ BC xenograft models, including those with PIK3CA mutations [44]. A multicenter phase 1b clinical trial evaluated the use of alpelisib plus fulvestrant in PIK3CA-altered and PIK3CA WT HR+ MBC progressing to antiestrogen therapy [45]. A total of 87 postmenopausal women were enrolled. The primary end point was the determination of the maximum tolerated dose (MTD). Secondary end points included safety and preliminary activity. The MTD of alpelisib plus fulvestrant was 400 mg once daily, and the recommended phase 2 dose was 300 mg once daily. Overall, the most frequent grade 3–4 AEs were hyperglycemia (22%) and maculopapular rash (13%). The median PFS at the MTD was 5.4 months and the median PFS with alpelisib favored patients with PIK3CA-altered tumors versus WT ones (9.1 versus 4.7 months, respectively, *p* not available). The ORR was 29% versus zero in the PIK3CA-altered group and in the WT one, respectively. Based on these positive results, a subsequent phase III study aimed to compare fulvestrant plus alpelisib (300 mg/day versus fulvestrant plus placebo in the same setting (SOLAR-1 study) (Table 1) [46]. PIK3CA mutations were detected in tumor tissue, and the primary endpoint of the study was PFS. The study is still ongoing; however, during the European Society of Medical Oncology (ESMO) Congress 2018, preliminary results showed that PFS was nearly doubled in patients with PIK3CA mutations who received alpelisib (11.0 versus 5.7 months for the experimental and placebo arms, respectively, *p* = 0.00065). Taselisib, another PI3K inhibitor with improved selectivity against mutated p110α, was tested in a phase II single-arm study in which 60 postmenopausal patients with HR+/HER2− MBC progressing to endocrine therapy were randomized to receive taselisib plus fulvestrant [47]. The primary endpoint was clinical activity that was confirmed among patients regardless of their PIK3CA mutational status (best confirmed response rate in patients with PIK3CA mutations: 38.5% and CBR: 38.5%; best confirmed response rate in patients PIK3CA WT: 14.3% and CBR: 23.8%; best confirmed response rate in patients with unknown PIK3CA mutation status: 20.0% and CBR: 30.0%). The effectiveness of taselisib plus fulvestrant in PI3KCA mutant tumors was further demonstrated in the SANDPIPER trial, a phase III double-blind randomized, placebo-controlled study, which enrolled pretreated HR+/HER2− MBC patients (Table 1) [48]. The trial met its primary endpoint, since PFS was longer in patients with PIK3CA-mutant tumors treated with taselisib plus fulvestrant (7.4 versus 5.4 months for the experimental and control arms, respectively, *p* = 0.0037). Survival data are still immature. The most frequent AEs in the experimental arm were diarrhea (grade 3–4: 12%) and hyperglycemia (grade 3–4: 11%); severe AEs were reported in 32% of patients. A recent study presented at the ASCO 2019 Annual Meeting, the FAKTION trial, tested a new AKT inhibitor, capivasertib [49]. This is a phase II study, with an initial dose escalation phase I part and a subsequent double-blind, randomized, controlled phase II. It enrolled 140 postmenopausal women with HR+/HER2− MBC who had not received more than three previous lines of endocrine treatment and up to one line of chemotherapy for metastatic disease; they were also required to have progressive disease during AI. Following the dose escalation in stage 1, patients were randomized to receive fulvestrant plus capivasertib or placebo. The study met its primary endpoint of PFS that was longer in the experimental arm (10.3 versus 4.8 months for the experimental and control arms, respectively, *p* = 0.004). Survival data are still immature (37% of maturity), but a preliminary data analysis seems to favor the capivasertib arm (26.0 versus 20.0 months, *p* = 0.007). PFS was also evaluated based on PI3K/AKT/PTEN pathway activation (hotspot PIK3CA mutation and PTEN by immunohistochemistry). In the activated group, PFS was 9.5 versus 5.2 months (*p* = 0.064), whereas in the non-activated group, PFS was 10.3 versus 4.8 months (*p* = 0.035) in the experimental and placebo arms, respectively [49].

#### 3.2.3. Mechanisms of Endocrine Resistance in PI3K/AKT Pathway

Despite the efficacy of PI3K-inhibitors in HR+/HER2− MBC that developed resistance to endocrine therapy, in particular, in the presence of the PI3K mutation, some patients still do not respond to these treatments or develop early disease progression [38]. Resistance to PI3K inhibitors is mainly associated with hyper-expression of the phosphatase and tensin homolog PTEN, which is a tumor suppressor protein that is lost or mutated in about 30% of human cancers. PTEN is a protein that antagonizes PI3K by catalyzing the de-phosphorylation of phosphatidylinositol 3,4,5-trisphosphate (PI3,4,5P3), which is the product of activated PI3K [50]. Therefore, PTEN acts to attenuate PI3K signaling and switch off proliferative and anti-apoptotic signals [38]. PTEN activity is upregulated by PIK3R1 (p85α regulatory subunit), which inhibits the catalytic activity of p110α and prevents PTEN ubiquitination, increasing its stability [51]. Inositol polyphosphate 4-phosphatase type II (INPP4B) is another protein that inhibits PI3K activity by catalyzing the de-phosphorylation of PIP2 [36]. INPP4B is a tumor suppressor and downregulates PTEN; however, it has been demonstrated that its upregulation could provide cancer development through the activation of AKT and SGK3 [52]. In fact, these proteins are responsible for the regulation of the cell cycle, and mutations of PTEN, PIK3R1, or INPP4B may be the cause of resistance to PI3K inhibitors.

## 4. CDK4/6 Inhibitors

### 4.1. Biological Functions and Cross-Link Interactions

CDK4 and CDK6 are enzymes that, together with D-type cyclins (D1, D2, D3), promote cell progression from the G1 to the S phase [53]. In the presence of mitogenic signals, these kinases form D-CDK4/6 complexes which phosphorylate RB, a tumor suppressor gene. This causes the repression of E2F transcription, which modulates the expression of a host of genes responsible for the coordination of subsequent cell cycle progression (cyclin E and cyclin A), DNA replication (MCM7 and PCNA), and mitotic progression (cyclin B1 and Cdk1). Cancer-specific mutations, such as those affecting receptor tyrosine kinases (RTKs), or RAS, RAF, PI3K, or PTEN mutations, can enhance cyclin D-dependent CDK4/6 activity [54]. Instead, cell type-specific RTK, RAF/MEK/ERK, and PI3K/AKT inhibitors, some hormone or interleukin antagonists, and anti-proliferative cytokines, such as transforming growth factor beta (TGF-β), can increase the threshold for CDK4/6 activation and synergize with CDK4/6 inhibitors to induce G1 phase cell cycle arrest. However, the mitogenic signaling elicited by D-CDK4/6 complexes is limited and down-regulated by a highly specific 16 kDa polypeptide inhibitor of CDK4, encoded by the *INK4a* gene [55]. In this phase, p16INK4a binds to cyclin D–CDK4, inhibiting its kinase activity, preventing RB phosphorylation, and arresting cells in the G1 phase of the cell cycle. Importantly, cells lacking functional RB are resistant to p16INK4a-mediated cell cycle arrest, implying that the ability of CDK4 and CDK6 to drive G1 phase progression requires RB [56]. Many studies identified p16INK4a as a frequent target of inactivating mutations and deletions in many human cancers and revealed that the loss-of-function of p16INK4a and RB generally occur as mutually exclusive events in tumor cells [54,56]. Therefore, the loss of RB expression induces high-levels of endogenous CKD4/6 inhibitors such as p16INK4a [57]. For this reason, tumor with high levels of p16ink4a, like human papilloma virus-positive cancers (e.g., cervical cancer and squamous cell cancer of the head and neck district), would be resistant to CDK4/6 inhibitors [58]. In HR+/HER2− BC, genetic loss of RB is rare (<3%), whereas cyclin D1 is overexpressed or amplified in a significant proportion of cases [59]. 

### 4.2. Clinical Trials

#### 4.2.1. Palbociclib

The first CDK4/6 selective inhibitor to be studied back in 2005 was palbociclib. In preclinical trials, palbociclib showed high efficacy in breast, colon, and lung cancer xenograft models, arresting the proliferation of tumor cell lines that retained functional RB [60]. Preclinical data also demonstrated the major sensitivity of ER + BC cell lines compared with ER− cell lines, probably due to a higher incidence of loss of RB function in this BC subgroup [61]. A subsequent phase I study demonstrated that treatment with 125 mg/day palbociclib for three weeks on a one week on/off schedule could be combined with the standard dose of letrozole (2.5 mg once daily) [54]. The PALOMA-1/TRIO-18 trial, an open label, phase II, double-blind study randomized study, involved 1:1 postmenopausal, treatment-naïve women with HR+/HER2− MBC who received palbociclib plus letrozole or letrozole alone (Table 2) [62]. Patients were recruited in two separate cohorts that accrued sequentially: cohort 1 enrolled patients only on the basis of their HR+/HER− status, whereas cohort 2 required patients to have tumors with cyclin D1 (CCND1) amplification, loss of p16 (INK4A or CDKN2A), or both. The study included 165 patients: 84 received palbociclib plus letrozole and 81 received letrozole alone. The study met its primary endpoint, since PFS was higher in the palbociclib plus letrozole group (20.2 versus 10.2 months for the experimental and placebo arms, respectively, *p* = 0.0004). In cohort 1 the median PFS favored the experimental arm with 26.1 versus 5.7 months (*p* < 0.0001); in cohort 2, the difference in PFS between the two arms was also evident but was reduced (18.1 versus 11.1 months for the palbociclib group and letrozole group alone, respectively). The most frequent grade 3–4 AEs in the palbociclib arm were neutropenia (54%) and leucopenia (19%). The subsequent double-blind, phase III, randomized study, the PALOMA-2 trial, enrolled 666 postmenopausal women with HR+/HER2− MBC who had not received prior treatment for advanced disease (Table 2) [63]. Patients were randomized 1:1 to receive either palbociclib plus letrozole or letrozole alone. The primary endpoint was PFS and it favored the experimental arm (24.8 versus 14.5 months, respectively, *p* < 0.001). The study confirmed that grade 3–4 neutropenia was the most common AE during treatment with palbociclib in all subgroups, but a downward trend in the severity of the neutropenia was shown over time after six cycles of treatment. Finally, the PALOMA-3 trial was a double-blind, randomized, phase III study which recruited HR+/HER2− MBC women, of both pre- and postmenopausal status, who relapsed or progressed during or after endocrine therapy, to receive palbociclib plus fulvestrant versus fulvestrant plus placebo (Table 2) [64]. The primary endpoint was PFS. The trial also assessed endocrine therapy resistance by clinical parameters, quantitative hormone receptor expression, and tumor PIK3CA mutational status in circulating DNA at baseline. A total of 521 patients were assigned to the treatment arm in a 2:1 ratio. The primary endpoint was met with a PFS that favored the palbociclib arm (9.5 versus 4.6 months for the experimental and placebo arms, respectively, *p* < 0.0001). The most common grade 3–4 AEs in the palbociclib arm were neutropenia (65%), anemia (3%), and leucopenia (28%). PIK3CA mutation was detected in the plasma DNA of 33% of patients; however, neither PIK3CA status nor the hormone-receptor expression level significantly affected the treatment response. Recently, a new analysis assessed OS, a key secondary endpoint of PALOMA-3, at a median follow-up time of 44.8 months [65]. Among the 521 patients who underwent randomization, the median OS was 34.9 versus 28.0 months (*p* = 0.09) in the palbociclib–fulvestrant group and in the placebo–fulvestrant group, respectively. However, despite the combination treatment resulting in a longer OS, the differences in OS in the entire trial group were not statistically significant.

#### 4.2.2. Ribociclib

Ribociclib is another orally bioavailable selective CDK4/6 inhibitor that was developed and approved for use in daily clinical practice. The drug showed its acceptable safety profile and activity in combination with letrozole in a phase 1b study that recruited women with HR+/HER2− MBC who had received no previous systemic treatment for advanced disease [66]. The trial showed an ORR of 46% and a CBR of 79% among patients with measurable disease. In a phase III, placebo-controlled study, ribociclib plus letrozole was administered as first line treatment in postmenopausal women with HR+/HER2− MBC (MONALEESA-2) (Table 2) [67]. A total of 668 patients were enrolled. The study met its primary endpoint of PFS favoring the experimental arm (25.3 versus 16.0 months, respectively, *p* < 0.001). Also, the ORR was higher in the ribociclib arm (42.5% versus 28.7%, respectively). Nausea, vomiting, alopecia, and diarrhea were the most frequent AEs in the ribociclib arm. These positive results were also maintained in pre-planned subgroups of elderly and younger patients. The subsequent phase III study, the MONALEESA-3, was a placebo-controlled, randomized clinical trial in which HR+/HER2− postmenopausal MBC women who were treatment naïve or had received up to one line of prior endocrine therapy in the advanced setting were randomized 2:1 to receive ribociclib plus fulvestrant versus placebo plus fulvestrant (Table 2) [68]. A total of 484 patients were assigned to the experimental arm, and 242 were assigned to the placebo one. The median PFS was significantly improved with ribociclib (20.5 versus 12.8 months, *p* < 0.001). In patients with measurable disease, the ORR favored the ribociclib arm (40.9% versus 28.7%, respectively). The most common AEs were neutropenia (46.6%), and leucopenia (13.5%). MONALEESA-7 was the first trial to focus on premenopausal, treatment-naïve women with HR+/HER− MBC [69]. Patients were randomly assigned to receive ribociclib or placebo plus goserelin and one of the following endocrine therapies: the nonsteroidal aromatase inhibitors letrozole or anastrozole, or tamoxifen (Table 2). PFS was the primary endpoint of the study and was found to be higher in the experimental arm (23.8 versus 13.0 months, *p* < 0.0001). The most common grade 3–4 AEs in the ribociclib arm were neutropenia (61%) and leucopenia (14%). A recent update of the prespecified interim analysis of OS was published, and it confirmed the superiority of the experimental arm (not reached versus 40.9 months, *p* = 0.00973) [70]. The PFS benefit was similar in the low and high gene expression subgroups, but a trend toward a PFS benefit with ribociclib in the presence of high cyclin D1, insulin-like growth factor-1 receptor (IGF1R), and erb-B2 receptor tyrosine kinase-3 (ERBB3) was reported. A stronger PFS benefit was reported with ribociclib in the presence of low cyclin E1 and MYC; finally, a trend toward a similar PFS benefit was observed with ribociclib, regardless of the lower and higher expression of ESR1, the value of the marker of proliferation, Ki67 (MKI67), and the FGFR1 gain. Interestingly, the TRINITI-1 trial was the first study to analyze the combination between CDK4/6 inhibitors, endocrine therapy, and mTOR inhibitors following progression with a CDK4/6 inhibitor [71]. This was a phase I/II, open-label trial of triplet therapy: ribociclib plus everolimus plus exemestane in men or postmenopausal women with HR+/HER2− MBC that progressed following CDK4/6 inhibitor therapy. The trial showed encouraging results, since the majority of patients had stable disease (49.5%) with a median PFS of 5.7 months. The ctDNA genotyping analyses revealed that patients with ESR1 or PIK3CA mutations had numerically shorter median PFS than those who were WT (6.9 versus 3.5 months for the ESR1 WT and ESR1 mutated patients, respectively; 7.3 versus 5.7 months for the PIK3CA WT and PIK3CA-mutated patients, respectively). These biomarker analyses, however, were hypothesis generating and need validation. In particular, the shorter PFS in patients with ctDNA mutation may reflect the presence of a more aggressive tumor or a higher tumor burden.

#### 4.2.3. Abemaciclib

Abemaciclib was the last CDK4/6 to be approved for clinical practice. In comparison with palbociclib and ribociclib, abemaciclib has a higher potency and greater specificity for CDK4 based on preclinical pharmacokinetic models [72]. The drug was first evaluated in a phase I study which established the maximum tolerated dose (MTD) for abemaciclib as a single agent as 200 mg/day every 12 h and demonstrated its safety and clinical activity against different human tumors including BC, lung cancer, melanoma, and mantle cell lymphoma [73]. In particular, in the hormone refractory HR+/HER2− MBC setting, the ORR was 26%. MONARCH-1 was a phase II, single arm, open-label study that enrolled 132 women with HR+/HER2− MBC who developed progressive disease during or after prior endocrine therapy and who had already undergone one or two chemotherapy regimens in the metastatic setting (Table 2) [74]. The primary objective was an ORR of 19.7% at the 12-month final analysis; among the secondary endpoints, the CBR (complete response + partial response + stable disease ≥6 months) was 42.4%, the median PFS was 6.0 months, and the median OS was 17.7 months. The most common AEs of any grade in the abemaciclib arm were diarrhea, fatigue, and nausea. Due to these promising clinical results, a subsequent phase III study was performed, the MONARCH-2 trial [75]. This double-blind study enrolled 669 women with HR+/HER2− MBC whose disease had progressed while receiving (neo)adjuvant endocrine therapy (≤12 months from the end of adjuvant endocrine therapy) or while receiving first-line endocrine therapy for metastatic disease. Patients were randomized 2:1 to receive abemaciclib plus fulvestrant or placebo plus fulvestrant. PFS was significantly higher in the experimental arm (16.4 versus 9.3 months, *p* < 0.001). In patients with measurable disease, abemaciclib group reached a higher ORR (48.1% versus 21.3%). The most common AEs in the abemaciclib arm were diarrhea (86.4%), neutropenia (46.0%), nausea (45.1%), and fatigue (39.9%). Meanwhile, abemaciclib was also tested in the first line metastatic setting in the MONARCH-3 trial (Table 2) [76]. This was a randomized, double-blind, phase III study that enrolled postmenopausal, treatment-naïve HR+/HER2− MBC patients to receive abemaciclib plus a non-steroidal AI versus a placebo plus a non-steroidal AI. A total of 493 women were enrolled, and the primary endpoint was PFS. In the final analysis, which was published after 240 events, the abemaciclib arm had a significantly higher PFS (28.18 versus 14.76 months; *p*  =  0.000002) [77]. Also, the ORR was higher in the experimental arm in patients with measurable disease (61.0% versus 45.5%, *p* = 0.003) as well as the median duration of response (27.39 versus 17.46 months). The most common grade  ≥3 AEs in the abemaciclib group were neutropenia (23.9%), diarrhea (9.5%), and leukopenia (8.6%). Since abemaciclib is the only CK4/6 inhibitor to cross the blood–brain barrier, a study recently presented at the ASCO 2019 Annual Meeting described abemaciclib activity in 58 treatment-naïve MBC patients with brain metastasis [78]. Patients had more than one new or previously not treated brain metastasis with a maximum diameter of more than 10 mm or a progressive, previously irradiated brain metastasis. The primary endpoint was the objective intracranial response rate (OIRR). The trial result was negative, since the predictive cut-off for the primary endpoint was 11% and the study obtained an OIRR of 6%, with a median treatment duration of 3.1 months. However, about 60% of patients obtained a stable disease lasting more than 6 months in about 20% of patients. The OIRR was also higher (29%) in patients who had not received local treatment (stereotactic radiotherapy or whole brain radiotherapy). Several clinical trials are trying to investigate the role of immunotherapy in endocrine sensitive MBC [79]. The inhibition of CD4/6-Rb-E2F induces tumor cell-cycle arrest and promotes an anti-tumor immune response through antigen presentation in tumor cells, T-cell activation, and suppression of the proliferation of regulatory T-cells (Tregs) [79]. An in vivo study by Goel and colleagues demonstrated that treatment with abemaciclib plus an anti-PD-L1 immunotherapy were effective in RB-competent BC cells due to the enhancement of both immunomodulatory and cell cycle suppression effects [80].

### 4.3. Mechanisms of Endocrine Resistance in the CDK 4/6 Pathway

The aforementioned trial showed the superiority of CDK 4/6 inhibitors plus an endocrine therapy over an endocrine treatment alone in different settings of HR+/HER− MBC. However, these drugs have different safety and pharmacokinetic profiles and, to date, no clinical or biological biomarkers exist in order to help clinicians to choose the best treatment for every patient. High p16ink4a expression or loss of RB are the most studied markers [81]. The biomarker analysis in PALOMA-2 did not show any correlation between the expression levels of RB, Ki67, cyclin D1, or p16 and the response to palbociclib plus letrozole [61], so the basis of the acquired cell resistance to CDK4/6 inhibitors remains largely unknown. Preclinical studies have suggested that resistance to CDK4/6 inhibitors could develop through RB1 mutation, cyclin E amplification, CDK6 amplification, or activation of CDK2 [82,83]. Intrinsic resistance to CDK 4/6 inhibitors has been described in the presence of RB loss-of-function and proto-cadherin FAT1 loss-of-function [84]. Herrera-Abreu and colleagues reported that chronic inhibition by CDK4/6 inhibitors was associated with increased AKT phosphorylation, which correlated with the sustained expression of E2F-induced G1-S phase regulators, such as cyclin E2 or CDK2, preventing the inhibition of RB phosphorylation [85]. In another study, Yang and colleagues described how some cell lines previously exposed to abemaciclib acquired amplification of CDK6 kinase with a reduced response to abemaciclib [86]. Moreover, CDK6 overexpression led to reduced expression of ER and PgR with decreased hormone-responsiveness. Since RB loss can occur more frequently along with metastatic progression, Condorelli and colleagues reported that MBC cells can acquire a polyclonal RB1 mutation after exposure to CDK4/6 inhibitors, which become ineffective [87]. From a recent update of the PALOMA 3 trial, some prognostic markers of early progression (without interaction with palbociclib) were described: a circulating tumor fraction of >10%, FGFR1 gain, and TP53 mutation in ctDNA [4]. 

## 5. Conclusions

Endocrine treatment is a mainstay for HR+/HER2− MBC [2]; however, endocrine resistance remains an important challenge. In the last few years, improved knowledge of the mechanism behind hormone resistance has allowed the development of new generations of targeted therapies for MBC treatment [11,79]. While PI3K/AKT/mTOR signaling is known to be an important growth pathway in HR+/HER− MBC, pan-PI3K inhibitors have shown disappointing results due to their modest effect sizes and significant toxicity [41,42]. On the other hand, the more selective PI3K inhibitors, such as alpelisib and taselisib, featured promising results, in particular, in PIK3CA-mutated cancer patients [46,48]. To date, only alpelisib has been approved by the Food and Drug Administration (FDA) in combination with fulvestrant for the treatment of HR+/HER2− MBC following progression on or after an endocrine-based regimen. The mTOR inhibitor everolimus significantly improves PFS when added to endocrine therapy, and everolimus plus exemestane remains a valid second/third line option after progression on hormonal therapy (tamoxifen/AI/fulvestrant with or without a CDK4/6 inhibitor) [22]. Recently, the use of selective CDK4/6 inhibitors, such as palbociclib, abemaciclib, and ribociclib, combined with endocrine agents, led to substantial PFS improvements in first- and second-line settings and changed the current clinical practice, becoming the treatment of choice in HR+/HER2− treatment-naïve or hormone pre-treated MBC patients without visceral crisis [2]. While these combinations of targeted therapies improve outcomes and often delay the initiation of chemotherapy, long term OS results remain scarce. Therefore, many issues are still to be addressed in the near future, as follows: (1) we are currently not aware of how cancer cells develop resistance to CDK4/6 and mTOR inhibitors; therefore, the best treatment after progression on these drugs is still unknown; (2) the best sequencing of all available treatment options still needs to be identified; and (3) how combinations of CDK4/6 inhibitors plus endocrine therapy and mTOR inhibitors plus endocrine therapy work compared with each other and with chemotherapy remains unknown. Further, studies should also be focused on the identification of reliable response and resistance biomarkers in order to select patients who could benefit most from the available therapies to avoid unnecessary side effects, “financial toxicity”, and, more importantly, to further improve the survival time and quality of life of HR+/HER2− MBC patients. 

## Figures and Tables

**Table 1:** Pivotal trials on the phosphatidylinositol 3-kinase (PI3K)/protein kinase B (AKT)/mammalian target of rapamycin (mTOR) pathway.

Author, Year [Ref]	Trial	Study Design	N° Patients	Treatment Line	Drug	Primary Endpoints	Results
Bachelot, 2012 [23]	TAMRAD	Phase II Randomized 1:1	111	Progressed on previous ET	EVE plus TAM versus TAM	CBR	61.1% versus 42.1%
Yardley, 2013 [22]	BOLERO-2	Phase III Placebo-controlled Double-blind Randomized 2:1	724	Progressed on previous ET	EVE plus EXE versus PBO plus EXE	PFS	11.0 versus 4.1 mo, *p* < 0.0001
Baselga, 2017 [40]	BELLE-2	Phase III Placebo-controlled Double-blind Randomized 1:1	1147	Progressed on previous ET	BUP plus F500 versus PBO plus F500	PFS (overall population and inactivated or non-activated PI3K pathway)	Overall population (*n* = 1147) 6.9 versus 5.0 mo, *p* = 0.00021; PIK3CA mutant (*n* = 200) 7.0 versus 3.2 mo, *p* = 0.0005 PIK3CA WT (*n* = 387) 6.8 versus 6.8 mo, *p* = 0.642 PI3K activated (*n* = 372) 6.8 versus 4.0 mo, *p* = 0.014 PI3K non-activated (*n* = 479) NR
Krop, 2016 [42]	FERGI	Phase II Placebo-controlled Double-blind Randomized: 1:1 (part 1) 2:1 (part 2)	168 (part 1) 61 (part 2)	Progressed on previous ET	PIC plus F500 versus PBO plus F500	PFS (overall population and in patients with PI3K mutated tumors)	Part 1 PIK3CA mutant (*n* = 70) 6.5 versus 5.1 mo, *p* = 0.268 PIK3CA WT (*n* = 84) 5.8 versus 3.6 mo, *p* = 0.23 Part 2 PIK3CA mutant (*n* = 61) 5.4 versus 10.0 mo, *p* = 0.84
Andrè, 2018 [46]	SOLAR-1	Phase III Double-blind Placebo-controlled Randomized 1:1	572	Progressed on previous ET	ALP plus F500 versus PBO plus F500	PFS	PIK3CA-mutated (*n* = 341) 11.0 versus 5.7 mo, *p* < 0.001
Baselga, 2018 [48]	SANDPIPER	Phase III Double-blind Placebo-controlled Randomized 2:1	631	Progressed on previous ET	TAS plus F500 versus PBO plus F500	PFS	7.4 versus 5.4 mo, *p* = 0.0037

Abbreviations: ALP: alpelisib; BUP: buparlisib; CBR: clinical benefit rate; ET: endocrine therapy; EXE: exemestane; EVE: everolimus; F500: fulvestrant; HR: hazard ratio; mo: months; N: number; NSAIs: nonsteroidal aromatase inhibitors; NR: not reported; PBO: placebo; PFS: progression-free survival; PIC: pictlisib; TAM: tamoxifen; TAS: taselisib; WT: wild-type.

**Table 2:** Pivotal trials on the cyclin-dependent kinase (CDK)4/6 pathway.

Author, Year [Ref]	Trial	Study Design	N° Patients	Treatment Line	Drug	Primary Endpoints	Results
Finn, 2015 [62]	PALOMA-1/ TRIO-18	Phase II Open-label Randomized 1:1	165	1° line	LET plus PAL versus LET	PFS	20.2 versus 10.2 mo; *p* = 0.0004
Finn, 2016 [63]	PALOMA-2	Phase III Placebo-controlled Double-blind Randomized 2:1	666	1° line	LET plus PAL versus LET plus PBO	PFS	24.8 versus 14.5 mo; *p* < 0.001
Cristofanilli, 2016 [64]	PALOMA-3	Phase III Placebo-controlled Double-blind Randomized 2:1	521	Progressed on previous ET	F500 +/-LHRH analogue plus PAL versus F500 +/-LHRH analogue plus PBO	PFS	9.5 versus 4.6 mo; *p* < 0.0001
Hortobagyi, 2017 [67]	MONALEESA-2	Phase III Placebo-controlled Double-blind Randomized 1:1	668	1° line postmenopausal	LET plus RIB versus LET plus PBO	PFS	25.3 versus 16 mo; *p* < 0.001
Slamon, 2018 [68]	MONALEESA-3	Phase III Placebo-controlled Double-blind Randomized 2:1	726	Progressed on previous ET	F500 plus RIB versus F500 plus PBO	PFS	20.5 versus 12.8 mo; *p* < 0.001
Tripathy, 2018 [69]	MONALEESA-7	Phase III Placebo-controlled Double-blind Randomized 1:1	672	1° line premenopausal	TAM/LET/ANA plus LHRH analogue plus RIB versus TAM/LET/ANA plus LHRH analogue plus PBO	PFS	23.8 versus 13 mo; *p* < 0.0001
Dickler, 2017 [74]	MONARCH-1	Phase II Single agent Open-label	132	Progressed on previous ET	ABE	ORR	CR 0 PR 17.4 % SD 40.2 % PD 25.0%
Goetz, 2017 [76]	MONARCH-3	Phase III Placebo-controlled Double-blind Randomized 2:1	493	1° line	LET or ANA plus ABE versus LET or ANA plus PBO	PFS	28.18 versus 14.76 mo; *p* = 0.000002

Abbreviations: ABE: abemaciclib; ANA: anastrozole; CR: complete response; F500: fulvestrant; LET: letrozole; HR: hazard ratio; mo: months; N: number; NR: not reached; ORR: overall response rate, PAL: palbociclib, PD: progressive disease; PFS: progression- free survival; PR: partial response; PBO: placebo; RIB: ribociclib; SD: stable disease; TAM: tamoxifen.

## References

[1] Bray F., Ferlay J., Soerjomataram I., Siegel R.L., Torre L.A., Jemal A. (2018). Global cancer statistics 2018: GLOBOCAN estimates of incidence and mortality worldwide for 36 cancers in 185 countries. CA Cancer J. Clin..

[2] Cardoso F., Senkus E., Costa A., Papadopoulos E., Aapro M., André F., Harbeck N., Aguilar Lopez B., Barrios C.H., Bergh J. (2018). 4th ESO–ESMO International Consensus Guidelines for Advanced Breast Cancer (ABC 4)†. Ann. Oncol..

[3] Chen K., Quan J., Yang J., Chen Z. (2019). The potential markers of endocrine resistance among HR+ /HER2+ breast cancer patients. Clin. Transl. Oncol..

[4] O’Leary B., Cutts R., Huang X., Hrebien S., Liu Y., Garcia-Murillas I., Andre F., Loi S., Loibl S., Cristofanilli M. (2019). Genomic markers of early progression on fulvestrant with or without palbociclib for ER+ advanced breast cancer in the PALOMA-3 trial. J. Clin. Oncol..

[5] Osborne C.K., Schiff R. (2011). Mechanisms of endocrine resistance in breast cancer. Annu. Rev. Med..

[6] Eyster K.M. (2016). The Estrogen Receptors: An Overview from Different Perspectives. Methods Mol. Biol..

[7] Arnal J.F., Fontaine C., Abot A., Valera M.C., Laurell H., Gourdy P., Lenfant F. (2013). Lessons from the dissection of the activation functions (AF-1 and AF-2) of the estrogen receptor alpha in vivo. Steroids.

[8] Bostner J., Skoog L., Fornander T., Nordenskjold B., Stal O. (2010). Estrogen receptor-alpha phosphorylation at serine 305, nuclear p21-activated kinase 1 expression, and response to tamoxifen in postmenopausal breast cancer. Clin. Cancer Res..

[9] Rani A., Stebbing J., Giamas G., Murphy J. (2019). Endocrine Resistance in Hormone Receptor Positive Breast Cancer-From Mechanism to Therapy. Front. Endocrinol..

[10] Ballinger T.J., Meier J.B., Jansen V.M. (2018). Current Landscape of Targeted Therapies for Hormone-Receptor Positive, HER2 Negative Metastatic Breast Cancer. Front. Oncol..

[11] Miller T.W., Rexer B.N., Garrett J.T., Arteaga C.L. (2011). Mutations in the phosphatidylinositol 3-kinase pathway: Role in tumor progression and therapeutic implications in breast cancer. Breast Cancer Res..

[12] Cortés J., Im S.A., Holgado E., Perez-Garcia J.M., Schmid P., Chavez-MacGregor M. (2017). The next era of treatment for hormone receptor-positive, HER2-negative advanced breast cancer: Triplet combination-based endocrine therapies. Cancer Treat. Rev..

[13] Lange C.A., Yee D. (2011). Killing the second messenger: Targeting loss of cell cycle control in endocrine-resistant breast cancer. Endocr. Relat. Cancer.

[14] Asghar U., Witkiewicz A.K., Turner N.C., Knudsen E.S. (2015). The history and future of targeting cyclin-dependent kinases in cancer therapy. Nat. Rev. Drug Discov..

[15] Jacquier M., Kuriakose S., Bhardwaj A., Zhang Y., Shrivastav A., Portet S., Varma Shrivastav S. (2018). Investigation of Novel Regulation of N-myristoyltransferase by Mammalian Target of Rapamycin in Breast Cancer Cells. Sci. Rep..

[16] Thangavel C., Dean J.L., Ertel A., Knudsen K.E., Aldaz C.M., Witkiewicz A.K., Clarke R., Knudsen E.S. (2011). Therapeutically activating RB: Reestablishing cell cycle control in endocrine therapy-resistant breast cancer. Endocr. Relat. Cancer.

[17] Pronzato P. (2017). Role of everolimus in the treatment of metastatic HER2-negative/HR-positive breast cancer. Future Oncol..

[18] Massacesi C., Di Tomaso E., Urban P., Germa C., Quadt C., Trandafir L., Aimone P., Fretault N., Dharan B., Tavorath R. (2016). PI3K inhibitors as new cancer therapeutics: Implications for clinical trial design. Onco Targets Ther..

[19] Spring L., Bardia A., Modi S. (2016). Targeting the cyclin D-cyclin-dependent kinase (CDK) 4/6-retinoblastoma pathway with selective CDK 4/6 inhibitors in hormone receptor-positive breast cancer: Rationale, current status, and future directions. Discov. Med..

[20] Baker S.J., Reddy E.P. (2012). CDK4. A Key Player in the Cell Cycle, Development, and Cancer. Genes Cancer.

[21] Dey N., De P., Leyland-Jones B. (2017). PI3K-AKT-mTOR inhibitors in breast cancers: From tumor cell signaling to clinical trials. Pharmacol. Ther..

[22] Yardley D.A., Noguchi S., Pritchard K.I., Burris H.A., Baselga J., Gnant M., Hortobagyi G.N., Campone M., Pistilli B., Piccart M. (2013). Everolimus plus exemestane in postmenopausal patients with HR(+) breast cancer: BOLERO-2 final progression-free survival analysis. Adv. Ther..

[23] Bachelot T., Bourgier C., Cropet C., Ray-Coquard I., Ferrero J.M., Freyer G., Abadie-Lacourtoisie S., Eymard J.C., Debled M., Spaëth D. (2012). Randomized phase II trial of everolimus in combination with tamoxifen in patients with hormone receptor-positive, human epidermal growth factor receptor 2-negative metastatic breast cancer with prior exposure to aromatase inhibitors: A GINECO study. J. Clin. Oncol..

[24] Piccart M., Hortobagyi G.N., Campone M., Pritchard K.I., Lebrun F., Ito Y., Noguchi S., Perez A., Rugo H.S., Deleu I. (2014). Everolimus plus exemestane for hormone-receptor-positive, human epidermal growth factor receptor-2-negative advanced breast cancer: Overall survival results from BOLERO-2. Ann. Oncol..

[25] Royce M., Bachelot T., Villanueva C., Özgüroglu M., Azevedo S.J., Cruz F.M., Debled M., Hegg R., Toyama T., Falkson C. (2018). Everolimus Plus Endocrine Therapy for Postmenopausal Women with Estrogen Receptor-Positive, Human Epidermal Growth Factor Receptor 2-Negative Advanced Breast Cancer: A Clinical Trial. JAMA Oncol..

[26] Chandarlapaty S., Chen D., He W., Sung P., Samoila A., You D., Bhatt T., Patel P., Voi M., Gnant M. (2016). Prevalence of ESR1 Mutations in Cell-Free DNA and Outcomes in Metastatic Breast Cancer: A Secondary Analysis of the BOLERO-2 Clinical Trial. JAMA Oncol..

[27] Cazzaniga M.E., Airoldi M., Arcangeli V., Artale S., Atzori F., Ballerio A., Bianchi G.V., Blasi L., Campidoglio S., Ciccarese M. (2017). On behalf of EVA Study Group. Efficacy and safety of Everolimus and Exemestane in hormone-receptor positive (HR+) human-epidermal-growth-factor negative (HER2-) advanced breast cancer patients: New insights beyond clinical trials. The EVA study. Breast.

[28] Schmid P., Zaiss M., Harper-Wynne C., Ferreira M., Dubey S., Chan S., Makris A., Nemsadze G., Brunt A.M., Kuemmel S. (2018). Abstract GS2-07 MANTA-A randomized phase II study of fulvestrant in combination with the dual mTOR inhibitor AZD2014 or everolimus or fulvestrant alone in estrogen receptor-positive advanced or metastatic breast cancer. Cancer Res..

[29] Faes S., Planche A., Uldry E., Santoro T., Pythoud C., Stehle J.C., Horlbeck J., Letovanec I., Riggi N., Dattaet D. (2016). Targeting carbonic anhydrase IX improves the anti-cancer efficacy of mTOR inhibitors. Oncotarget.

[30] Faes S., Demartines N., Dormond O. (2017). Resistance to mTORC1 Inhibitors in Cancer Therapy: From Kinase Mutations to Intratumoral Heterogeneity of Kinase Activity. Oxid. Med. Cell. Longev..

[31] De Iuliis F., Salerno G., Giuffrida A., Milana B., Taglieri L., Rubinacci G., Giantulli S., Terella F., Silvestri I., Scarpa S. (2016). Breast cancer cells respond differently to docetaxel depending on their phenotype and on survivin upregulation. Tumor Biol..

[32] Taglieri L., De Iuliis F., Giuffrida A., Giantulli S., Silvestri I., Scarpa S. (2017). Resistance to the mTOR inhibitor everolimus is reversed by the downregulation of survivin in breast cancer cells. Oncol. Lett..

[33] Bihani T., Ezell S.A., Ladd B., Grosskurth S.E., Mazzola A.M., Pietras M., Reimer C., Zinda M., Fawell S., D’Cruz C.M. (2014). Resistance to everolimus driven by epigenetic regulation of MYC in ER+ breast cancers. Oncotarget.

[34] Kimura M., Hanamura T., Tsuboi K., Kaneko Y., Yamaguchi Y., Niwa T., Narui K., Endo I., Hayashi S.I. (2018). Acquired resistance to everolimus in aromatase inhibitor-resistant breast cancer. Oncotarget.

[35] Vanhaesebroeck B., Guillermet-Guibert J., Graupera M., Bilanges B. (2010). The emerging mechanisms of isoform-specific PI3K signaling. Nat. Rev. Mol. Cell. Biol..

[36] Balselga J. (2011). Targeting the Phosphoinositide-3 (PI3) Kinase Pathway in Breast Cancer. Oncologist.

[37] Mukohara T. (2015). PI3K mutations in breast cancer: Prognostic and therapeutic implications. Breast Cancer (Dove Med Press).

[38] Ciruelos Gil E.V. (2014). Targeting the PI3K/AKT/mTOR pathway in estrogen receptor-positive breast cancer. Cancer Treat. Rev..

[39] Miller T.W., Hennessy B.T., González-Angulo A.M., Fox E.M., Mills G.B., Chen H., Higham C., García-Echeverría C., Shyr Y., Arteaga C.L. (2010). Hyperactivation of phosphatidylinositol-3 kinase promotes escape from hormone dependence in estrogen receptor-positive human breast cancer. J. Clin. Investig..

[40] Baselga J., Im S.A., Iwata H., Cortés J., De Laurentiis M., Jiang Z., Arteaga C.L., Jonat W., Clemons M., Ito Y. (2017). Buparlisib plus fulvestrant versus placebo plus fulvestrant in postmenopausal, hormone receptor-positive, HER2-negative, advanced breast cancer (BELLE-2): A randomised, double-blind, placebo-controlled, phase 3 trial. Lancet Oncol..

[41] Campone M., Im S.A., Iwata H., Clemons M., Ito Y., Awada A., Chia S., Jagiełło-Gruszfeld A., Pistilli B., Tseng L.M. (2018). Buparlisib plus fulvestrant versus placebo plus fulvestrant for postmenopausal, hormone receptor-positive, human epidermal growth factor receptor 2-negative, advanced breast cancer: Overall survival results from BELLE-2. Eur. J. Cancer.

[42] Krop I.E., Mayer I.A., Ganju V., Dickler M., Johnston S., Morales S., Yardley D.A., Melichar B., Forero-Torres A., Lee S.C. (2016). Pictilisib for oestrogen receptor-positive, aromatase inhibitor-resistant, advanced or metastatic breast cancer (FERGI): A randomised, double-blind, placebo-controlled, phase 2 trial. Lancet Oncol..

[43] Jia S., Roberts T.M., Zhao J.J. (2009). Should individual PI3 kinase isoforms be targeted in cancer?. Curr. Opin. Cell. Biol..

[44] Fritsch C., Huang A., Chatenay-Rivauday C., Schnell C., Reddy A., Liu M., Kauffmann A., Guthy D., Erdmann D., De Pover A. (2014). Characterization of the novel and specific PI3Kα inhibitor NVP-BYL719 and development of the patient stratification strategy for clinical trials. Mol. Cancer Ther..

[45] Juric D., Janku F., Rodón J., Burris H.A., Mayer I.A., Schuler M., Seggewiss-Bernhardt R., Gil-Martin M., Middleton M.R., Baselga J. (2019). Alpelisib Plus Fulvestrant in PIK3CA-Altered and PIK3CA-Wild-Type Estrogen Receptor–Positive Advanced Breast Cancer: A Phase 1b Clinical Trial. JAMA Oncol..

[46] André F., Ciruelos E.M., Rubovszky G., Campone M., Loibl S., Rugo H.S., Iwata H., Conte P., Mayer I.A., Kaufman B. Alpelisib (ALP) + fulvestrant (FUL) for advanced breast cancer (ABC): Results of the phase 3 SOLAR-1 trial. Proceedings of the ESMO 2018 Congress.

[47] Dickler M.N., Saura C., Richards D.A., Krop I.E., Cervantes A., Bedard P.L., Patel M.R., Pusztai L., Oliveira M., Cardenas A.K. (2018). A Phase II Study of Taselisib (GDC-0032) in Combination with Fulvestrant in Patients with HER2-Negative, Hormone Receptor–Positive Advanced Breast Cancer. Clin. Cancer Res..

[48] Baselga J., Dent S.F., Cortés J., Im Y.H., Diéras V., Harbeck N., Krop I.E., Verma S., Wilson T.R., Jin H. (2018). Phase III study of taselisib (GDC-0032) + fulvestrant (FULV) v FULV in patients (pts) with estrogen receptor (ER)-positive, PIK3CA-mutant (MUT), locally advanced or metastatic breast cancer (MBC): Primary analysis from SANDPIPER. J. Clin. Oncol..

[49] Jones R.H., Carucci M., Casbard A.C., Butler R., Alchami F., Bale C.J., Bezecny P., Joffe J., Moon S., Twelves C. (2019). Capivasertib (AZD5363) plus fulvestrant versus placebo plus fulvestrant after relapse or progression on an aromatase inhibitor in metastatic ER-positive breast cancer (FAKTION): A randomized, double-blind, placebo-controlled, phase II trial. J. Clin. Oncol..

[50] Gao X., Qin T., Mao J., Zhang J., Fan S., Lu Y., Sun Z., Zhang Q., Song B., Li L. (2019). PTENP1/miR-20a/PTEN axis contributes to breast cancer progression by regulating PTEN via PI3K/AKT pathway. J. Exp. Clin. Cancer Res..

[51] Chagpar R.B., Links P.H., Pastor M.C., Furber L.A., Hawrysh A.D., Chamberlain M.D., Andersonet D.H. (2010). Direct positive regulation of PTEN by the p85 subunit of phosphatidylinositol 3-kinase. Proc. Natl. Acad. Sci. USA..

[52] Guo S.T., Chi M.N., Yang R.H., Guo X.Y., Zan L.K., Wang C.Y., Xi Y.F., Jin L., Croft A., Tseng H.Y. (2016). INPP4B is an oncogenic regulator in human colon cancer. Oncogene.

[53] Knudsen E.S., Pruitt S.C., Hershberger P.A., Witkiewicz A.K., Goodrich D.W. (2019). Cell Cycle and Beyond: Exploiting New RB1 Controlled Mechanisms for Cancer Therapy. Trends Cancer.

[54] Sherr C.J., Beach D., Shapiro G.I. (2015). Targeting CDK4 and CDK6: From Discovery to Therapy. Cancer Discov..

[55] Peyressatre M., Prével C., Pellerano M., Morris M.C. (2015). Targeting Cyclin-Dependent Kinases in Human Cancers: From Small Molecules to Peptide Inhibitors. Cancers.

[56] Witkiewicz A.K., Knudsen E.S. (2014). Retinoblastoma tumor suppressor pathway in breast cancer: Prognosis, precision medicine, and therapeutic interventions. Breast Cancer Res..

[57] Liu Y., Zhong X., Wan S., Zhang W., Lin J., Zhang P., Li Y. (2014). p16INK4a expression in retinoblastoma: A marker of differentiation grade. Diagn. Pathol..

[58] Witkiewicz A.K., Knudsen K.E., Dicker A.P., Knudsen E.S. (2011). The meaning of p16(ink4a) expression in tumors: Functional significance, clinical associations and future developments. Cell Cycle.

[59] Bower J.J., Vance L.D., Psioda M., Smith-Roe S.L., Simpson D.A., Ibrahim J.G., Hoadley K.A., Perou C.M., Kaufmann W.K. (2013). Patterns of cell cycle checkpoint deregulation associated with intrinsic molecular subtypes of human breast cancer cells. Breast Cancer.

[60] Hamilton E., Infante J.R. (2016). Targeting CDK4/6 in patients with cancer. Cancer Treat. Rev..

[61] Finn R.S., Dering J., Conklin D., Kalous O., Cohen D.J., Desai A.J., Ginther C., Atefi M., Chen I., Fowst C. (2009). PD 0332991, a selective cyclin D kinase 4/6 inhibitor, preferentially inhibits proliferation of luminal estrogen receptor-positive human breast cancer cell lines in vitro. Breast Cancer Res..

[62] Finn R.S., Crown J.P., Lang I., Boer K., Bondarenko I.M., Kulyk S.O., Ettl J., Patel R., Pinter T., Schmidt M. (2015). The cyclin-dependent kinase 4/6 inhibitor palbociclib in combination with letrozole versus letrozole alone as first-line treatment of oestrogen receptor-positive, HER2-negative, advanced breast cancer (PALOMA-1/TRIO-18): A randomised phase 2 study. Lancet Oncol..

[63] Finn R.S., Martin M., Rugo H.S., Jones S., Im S.A., Gelmon K., Harbeck N., Lipatov O.N., Walshe J.M., Moulder S. (2016). Palbociclib and Letrozole in Advanced Breast Cancer. N. Engl. J. Med..

[64] Cristofanilli M., Turner N.C., Bondarenko I., Ro J., Im S.A., Masuda N., Colleoni M., DeMichele A., Loi S., Verma S. (2016). Fulvestrant plus palbociclib versus fulvestrant plus placebo for treatment of hormone-receptor-positive, HER2-negative metastatic breast cancer that progressed on previous endocrine therapy (PALOMA-3): Final analysis of the multicentre, double-blind, phase 3 randomised controlled trial. Lancet Oncol..

[65] Turner N.C., Slamon D.J., Ro J., Bondarenko I., Im S.A., Masuda N., Colleoni M., De Michele A., Loi S., Verma S. (2018). Overall Survival with Palbociclib and Fulvestrant in Advanced Breast Cancer. N. Engl. J. Med..

[66] Juric D., Munster P., Campone M., Ismail-Khan R., García-Estevez L., Hamilton E.P., Becerra C., De Boer R.H., Hui R., Goncalveset A. Ribociclib (LEE011) and letrozole in estrogen receptor-positive (ER+), HER2-negative (HER2–) advanced breast cancer (aBC): Phase Ib safety, preliminary efficacy and molecular analysis. Proceedings of the American Society of Clinical Oncology (ASCO) Annual Meeting.

[67] Hortobagyi G.N., Stemmer S.M., Burris H.A., Yap Y.S., Sonke G.S., Paluch-Shimon S., Campone M., Petrakova K., Blackwell K.L., Winer E.P. (2017). Updated results from MONALEESA-2, a phase 3 trial of first-line ribociclib + letrozole in hormone receptor-positive (HR+), HER2-negative (HER2–), advanced breast cancer (ABC). J. Clin. Oncol..

[68] Slamon D.J., Neven P., Chia S., Fasching P.A., De Laurentiis M., Im S.A., Petrakova K., Val Bianchi G., Esteva F.J., Martín M. (2018). Phase III Randomized Study of Ribociclib and Fulvestrant in Hormone Receptor–Positive, Human Epidermal Growth Factor Receptor 2–Negative Advanced Breast Cancer: MONALEESA-3. J. Clin. Oncol..

[69] Tripathy D., Im S.A., Colleoni M., Franke F., Bardia A., Harbeck N., Hurvitz S.A., Chow L., Sohn J., Lee K.S. (2018). Ribociclib plus endocrine therapy for premenopausal women with hormone-receptor-positive, advanced breast cancer (MONALEESA-7): A randomised phase 3 trial. Lancet Oncol..

[70] Im S.A., Lu Y.S., Bardia A., Harbeck N., Colleoni M., Franke F., Chow L., Sohn J., Lee K.S., Campos-Gomez S. (2019). Overall Survival with Ribociclib plus Endocrine Therapy in Breast Cancer. N. Engl. J. Med..

[71] Bardia A., Hurvitz S.A., DeMichele A., Clark A.S., Zelnak A.B., Yardley D.A., Karuturi M.S., Sanft T.B., Blau S., Hart L.L. (2019). Triplet therapy (continuous ribociclib, everolimus, exemestane) in HR+/HER2− advanced breast cancer postprogression on a CDK4/6 inhibitor (TRINITI-1): Efficacy, safety, and biomarker results. J. Clin. Oncol..

[72] Lallena M.J., Boehnke K., Torres R., Hermoso A., Amat J., Calsina B., De Dios A., Buchanan S., Du J., Beckmann R.P. In-vitro characterization of Abemaciclib pharmacology in ER+ breast cancer cell lines. Proceedings of the 106th Annual Meeting of the American Association for Cancer Research.

[73] Patnaik A.R.L., Tolaney S.M., Tolcher A.W., Goldman J.W., Gandhi L., Papadopoulos K.P., Beeram M., Rasco D.W., Hilton J.F., Nasir A. (2016). Efficacy and Safety of Abemaciclib, an Inhibitor of CDK4 and CDK6, for Patients with Breast Cancer, Non–Small Cell Lung Cancer, and Other Solid Tumors. Cancer Discov..

[74] Dickler M.N., Tolaney S.M., Rugo H.S., Cortés J., Diéras V., Patt D., Wildiers H., Hudis C.A., O’Shaughnessy J., Zamora E. (2017). MONARCH 1, A Phase II Study of Abemaciclib, a CDK4 and CDK6 Inhibitor, as a Single Agent, in Patients with Refractory HR+/HER2- Metastatic Breast Cancer. Clin. Cancer Res..

[75] Sledge G.W., Toi M., Neven P., Sohn J., Inoue K., Pivot X., Burdaeva O., Okera M., Masuda N., Kaufman P.A. (2017). MONARCH 2: Abemaciclib in Combination With Fulvestrant in Women With HR+/HER2− Advanced Breast Cancer Who Had Progressed While Receiving Endocrine Therapy. J. Clin. Oncol..

[76] Goetz M.P., Toi M., Campone M., Sohn J., Paluch-Shimon S., Huober J., Park I.H., Trédan O., Chen S.C., Manso L. (2017). MONARCH 3: Abemaciclib as Initial Therapy for Advanced Breast Cancer. J. Clin. Oncol..

[77] Johnston S., Martin M., Di Leo A., Im S.A., Awada A., Forrester T., Frenzel M., Hardebeck M.C., Cox J., Barriga S. (2019). MONARCH 3 final PFS: A randomized study of abemaciclib as initial therapy for advanced breast cancer. NPJ Breast Cancer.

[78] Anders C.K., Le Rhun E., Bachelot T.D., Yardley D.A., Awada A., Conte P., Kabos P., Bear M., Yang Z., Chen Y. (2019). A phase II study of abemaciclib in patients (pts) with brain metastases (BM) secondary to HR+, HER2- metastatic breast cancer (MBC). J. Clin. Oncol..

[79] Ribnikar D., Volovat S.R., Cardoso F. (2019). Targeting CDK4/6 pathways and beyond in breast cancer. Breast.

[80] Goel S., DeCristo M.J., Watt A.C., BrinJones H., Sceneay J., Li B.B., Khan N., Ubellacker J.M., Xie S., Metzger-Filho O. (2017). CDK4/6 inhibition triggers anti-tumour immunity. Nature.

[81] Knudsen E.S., Witkiewicz A.K. (2017). The Strange Case of CDK4/6 Inhibitors: Mechanisms, Resistance, and Combination Strategies. Trends Cancer.

[82] Teh J.L.F., Aplin A.E. (2019). Arrested Developments: CDK4/6 Inhibitor Resistance and Alterations in the Tumor Immune Microenvironment. Clin Cancer Res..

[83] Li W., Kotoshiba S., Berthet C., Hilton M.B., Kaldis P. (2009). Rb/Cdk2/Cdk4 triple mutant mice elicit an alternative mechanism for regulation of the G1/S transition. Proc. Natl. Acad. Sci. USA.

[84] Razavi P., Henrique dos Anjos C., Brown D.N., Qing L., Ping C., Herbert J., Colon J., Liu D., Mao M., Norton L. (2019). Molecular profiling of ER+ metastatic breast cancers to reveal association of genomic alterations with acquired resistance to CDK4/6 inhibitors. J. Clin. Oncol..

[85] Herrera-Abreu M.T., Palafox M., Asghar U., Rivas M.A., Cutts R.J., Garcia-Murillas I., Pearson A., Guzman M., Rodriguez O., Grueso J. (2016). Early adaptation and acquired resistance to CDK4/6 inhibition in estrogen receptor-positive breast cancer. Cancer Res..

[86] Yang C., Li Z., Bhatt T., Dickler M., Giri D., Scaltriti M., Baselga J., Rosen N., Chandarlapaty S. (2017). Acquired CDK6 amplification promotes breast cancer resistance to CDK4/6 inhibitors and loss of ER signaling and dependence. Oncogene.

[87] Condorelli R., Spring L., O’Shaughnessy J., Lacroix L., Bailleux C., Scott V., Dubois J., Nagy R.J., Lanman R.B., Iafrate A.J. (2018). Polyclonal RB1 mutations and acquired resistance to CDK4/6 inhibitors in patients with metastatic breast cancer. Ann. Oncol..

